# Burnout Dimensions and Associated Factors Among Orthopedic and Traumatology Physicians: A Cross-Sectional Study from Türkiye

**DOI:** 10.3390/healthcare14172696

**Published:** 2026-08-24

**Authors:** Ahmet Yiğitbay, Muhammet Can Ari, Başak Özge Yiğitbay, Cemal Kural, Nezih Ziroğlu

**Affiliations:** 1 Department of Orthopedics and Traumatology, İzmir Tepecik Training and Research Hospital, University of Health Sciences, 35170 İzmir, Turkey; ahmetyigitbay@gmail.com; 2 Department of Orthopedics and Traumatology, Sakarya Training and Research Hospital, 54100 Sakarya, Turkey; drmcan.1992@gmail.com; 3 Department of Mental Health and Diseases, Mehmet Akif İnan Training and Research Hospital, 63040 Şanlıurfa, Turkey; drbasakozge@gmail.com; 4 Department of Orthopedics and Traumatology, Bakirkoy Dr. Sadi Konuk Training and Research Hospital, University of Health Sciences, 34147 Istanbul, Turkey; cemalkural@hotmail.com; 5 Department of Orthopedic Prosthetics and Orthotics, Vocational School of Health Services, Acibadem Mehmet Ali Aydinlar University, 34303 Istanbul, Turkey; 6 Department of Orthopaedics and Traumatology, Acibadem University Atakent Hospital, 34303 Istanbul, Turkey

**Keywords:** burnout, Maslach Burnout Inventory, orthopedic surgery, resident physicians, traumatology, Türkiye, workload

## Abstract

**Background/Objectives:** Burnout is an important occupational health concern among orthopedic and traumatology physicians. This study aimed to evaluate the individual dimensions of burnout and identify their demographic and occupational correlates among orthopedic and traumatology physicians practicing in Türkiye. **Methods:** This cross-sectional study used an anonymous online survey distributed through the Turkish Society of Orthopaedics and Traumatology electronic mailing group between October 2024 and June 2026. Burnout was assessed using the Turkish adaptation of the 22-item Maslach Burnout Inventory, comprising the Emotional Exhaustion (EE), Depersonalization (DP), and Personal Accomplishment (PA) subscales. Unadjusted group comparisons, Spearman correlation analyses, and separate multivariable linear regression models with HC3 heteroscedasticity-consistent standard errors were performed. **Results:** A total of 320 complete questionnaires were analyzed. The mean EE, DP, and PA scores were 20.17 ± 6.83, 7.40 ± 3.58, and 20.37 ± 4.19, respectively. The subscale scores were analyzed as continuous dimensions rather than classified using low-, moderate-, or high-burnout thresholds. In unadjusted comparisons, residents had higher EE and DP scores and lower PA scores than specialists. After adjustment, resident status remained associated with higher DP (B = 2.14, *p* = 0.010) and lower PA (B = −1.92, *p* = 0.018). Seeing more than 120 patients per day was associated with higher EE (B = 4.22, *p* = 0.023) and DP (B = 2.49, *p* = 0.018), while working more than 100 h per week was associated with higher EE (B = 4.47, *p* = 0.009). Individual predictor effect sizes and the explanatory power of the models were generally modest. **Conclusions:** Daily patient volume, weekly working hours, and professional status showed distinct associations with the individual dimensions of burnout. These findings suggest that workload organization and support for resident physicians warrant consideration. However, because of the cross-sectional design and voluntary non-probability sampling, the findings should not be interpreted as causal or nationally representative.

## 1. Introduction

Burnout is a work-related psychological syndrome arising in response to persistent occupational stressors. It is commonly conceptualized through three related but distinct dimensions: Emotional Exhaustion (EE), Depersonalization (DP), and reduced Personal Accomplishment (PA) [1,2]. Emotional Exhaustion reflects the depletion of emotional and physical resources, whereas Depersonalization refers to detached, cynical, or impersonal attitudes toward patients. Reduced Personal Accomplishment denotes diminished feelings of professional competence and effectiveness [1,2]. Because these dimensions represent different manifestations of occupational strain, evaluating them separately may provide more informative evidence than treating burnout as a single, uniform outcome.

Physician burnout is important not only because of its association with physicians’ mental health, well-being, and career satisfaction, but also because of its implications for the functioning and sustainability of healthcare organizations. A systematic review and meta-analysis of 170 observational studies found that physician burnout was associated with lower job satisfaction, greater career-choice regret, increased turnover intention, patient-safety incidents, reduced professionalism, and lower patient satisfaction [3]. Physicians experiencing burnout had more than three times the odds of intending to leave their jobs compared with physicians without burnout symptoms [3]. Consistent with these findings, a large multicenter study involving 5312 physicians and 15,738 nurses showed that clinician burnout was associated with frequent staff turnover and unfavorable assessments of patient safety and care quality [4]. These findings indicate that physician burnout should be considered not solely an individual mental-health concern, but also an organizational and patient-safety issue. Nevertheless, because most available studies are observational, the reported relationships should be interpreted as associations rather than evidence of direct causality [3,4].

Although burnout among physicians has been extensively investigated, its reported prevalence varies substantially across studies. A systematic review found estimates ranging from 0% to 80.5%, with marked variation in burnout definitions, measurement instruments, scoring thresholds, medical specialties, and study quality [5]. This methodological heterogeneity limits direct comparisons among studies and makes it difficult to transfer findings across specialties and national healthcare systems [3,5]. Furthermore, combining the MBI dimensions into a single dichotomous outcome may obscure the possibility that occupational and professional factors have different relationships with EE, DP, and PA [1,2]. Specialty- and context-specific studies that examine these dimensions separately are therefore necessary.

Orthopedic surgery and traumatology constitute a particularly demanding clinical environment. Physicians in this specialty must frequently combine high-volume outpatient services with emergency trauma management, complex surgical decision-making, prolonged operative procedures, overnight duties, and substantial physical demands [6,7,8]. A recent systematic review reported an overall burnout prevalence of approximately 48.9% among orthopedic surgeons, while also identifying considerable between-study variation [9]. Studies from Iran, France, and Jordan have similarly documented substantial burnout among orthopedic surgeons and residents, although the reported magnitude and dimensional pattern of burnout differed across settings [10,11,12]. These differences suggest that the presence of burnout in orthopedic practice is well established, whereas the independent contributions of specific workload, professional, and organizational factors remain less clearly resolved.

There are conceptual and empirical reasons to expect workload indicators to relate differently to the three burnout dimensions. High daily patient volume and prolonged weekly working hours may progressively deplete physicians’ emotional and physical resources and may therefore be most directly associated with EE [6,7,8,9,13]. Sustained patient contact under severe time pressure may also encourage psychological distancing as a coping response, potentially contributing to DP [1,2,13]. In a nationwide study of Ecuadorian physicians, long working hours were associated with greater EE, whereas shift work was associated with greater DP [13]. However, evidence concerning individual workload indicators is not entirely consistent. For example, a longitudinal pilot study of pharmacy residents found no statistically significant association between the number of required weekend staffing shifts and changes in burnout scores, although overall EE increased during the residency period [14]. Although pharmacy residents differ from orthopedic physicians in their professional responsibilities, this finding illustrates that working-time indicators may not capture the qualitative intensity, autonomy, responsibility, or clinical complexity of occupational demands. Workload should therefore be evaluated using multiple indicators, including patient volume, total weekly working hours, on-call duties, and standby duties.

Professional status may also have dimension-specific associations with burnout. Compared with specialists, resident physicians generally have less clinical experience and professional autonomy while simultaneously facing intensive training requirements, frequent on-call duties, performance evaluation, and substantial clinical responsibility. Reviews and comparative studies have reported that orthopedic residents may have less favorable burnout profiles than senior physicians or faculty members [15,16,17]. Residency status may be particularly relevant to DP and PA because limited autonomy, repeated evaluation, and uncertainty regarding professional competence may affect physicians’ interpersonal engagement and perceptions of professional effectiveness. Demographic and institutional characteristics may additionally influence burnout through differences in social support, available resources, professional autonomy, and exposure to occupational demands [3,9,13]. Examining professional status and workload variables simultaneously is therefore necessary to distinguish their independent relationships with each burnout dimension.

Evidence from Türkiye indicates that burnout is an important concern among healthcare professionals across different clinical settings [18]. However, findings obtained from heterogeneous groups of healthcare workers cannot fully represent the distinctive clinical, physical, and organizational demands experienced by orthopedic and traumatology physicians. Moreover, most specialty-specific orthopedic evidence has originated from other national healthcare systems and has employed heterogeneous burnout definitions and analytical approaches [9,10,11,12]. To our knowledge, nationwide evidence evaluating EE, DP, and PA specifically among orthopedic and traumatology physicians in Türkiye is lacking. It remains unclear whether professional status and potentially modifiable workload indicators—including daily patient volume, weekly working hours, on-call duties, and standby duties—are independently associated with the individual dimensions of burnout after demographic, institutional, and practice-related characteristics are considered. Although individual-focused interventions such as mindfulness, coaching, and peer support may reduce physician burnout symptoms, identifying upstream organizational correlates remains essential for developing interventions directed at working conditions rather than placing responsibility exclusively on individual physicians [19].

Accordingly, this study aimed to evaluate the three individual dimensions of burnout—Emotional Exhaustion (EE), Depersonalization (DP), and Personal Accomplishment (PA)—among orthopedic and traumatology resident and specialist physicians practicing in Türkiye and to determine whether professional status and occupational workload indicators were independently associated with these dimensions.

Two prespecified hypotheses were tested. First, we hypothesized that greater occupational workload, indicated by higher daily patient volume, longer weekly working hours, and more frequent monthly on-call and standby duties, would be associated with higher EE and DP scores and lower PA scores (H1). Second, we hypothesized that resident physicians would have higher EE and DP scores and lower PA scores than specialist physicians after adjustment for demographic, institutional, and workload-related characteristics (H2). Associations involving age, marital status, institution type, and practice location were examined as covariate-adjusted exploratory relationships because their direction and magnitude have not been consistent across previous studies.

## 2. Materials and Methods

### 2.1. Study Design and Rationale

This nationwide cross-sectional survey was conducted between October 2024 and June 2026 to evaluate the three dimensions of burnout and their demographic and occupational correlates among orthopedic and traumatology physicians in Türkiye. A cross-sectional design was selected because the primary objective was to assess Emotional Exhaustion (EE), Depersonalization (DP), and Personal Accomplishment (PA) scores and their contemporaneous relationships with multiple occupational characteristics in a geographically dispersed professional population. This design enabled the simultaneous assessment of professional status, institutional characteristics, workload indicators, and burnout dimensions without requiring longitudinal follow-up. However, because exposures and outcomes were measured at the same time, temporal sequence and causal relationships could not be established.

The online survey remained available throughout the extended 2024–2026 recruitment period to increase nationwide reach and facilitate voluntary participation by physicians with demanding and variable work schedules. No major nationwide healthcare policy change specifically targeting the employment or working arrangements of orthopedic and traumatology physicians occurred during this period. Nevertheless, local staffing levels, institutional practices, patient volumes, and individual working conditions may have changed over time. The extended data-collection period was therefore considered a potential source of temporal heterogeneity when interpreting the results.

### 2.2. Target Population and Recruitment

The target population consisted of orthopedic and traumatology specialists and resident physicians registered as members of the Turkish Society of Orthopaedics and Traumatology (TOTBİD). According to the TOTBİD 2025–2027 Strategic Plan, the society had 3108 full members, representing specialist physicians, and 1567 candidate members, representing physicians undergoing orthopedic and traumatology residency training, as of April 2026. The total target population therefore comprised 4675 physicians [20].

The study was not designed to obtain a probability-based or nationally representative sample, and no sample-size calculation for estimating population prevalence with a prespecified margin of error was performed. Instead, the adequacy of the achieved sample was evaluated in relation to the requirements of the planned multivariable regression analyses, as described in the Sample-Size Adequacy subsection. Accordingly, the findings should be interpreted as associations observed within the study sample rather than as nationally representative estimates.

Participants were recruited through voluntary self-selection from the national electronic mailing group operated by TOTBİD. An invitation explaining the purpose of the study and containing a link to the anonymous online questionnaire was distributed through this mailing group. No random, stratified, cluster-based, or institution-based selection procedure was applied. Physicians were not individually selected according to institution, geographic location, professional status, workload, or presumed level of burnout. Participation was voluntary, and no financial or professional incentive was provided. The resulting sample was therefore a voluntary non-probability sample.

The survey was intended for TOTBİD-member orthopedic and traumatology residents and specialists who were actively practicing in Türkiye during the study period. Access to the questionnaire required electronic informed consent. No additional exclusion criteria were applied after questionnaire completion. All 320 participants who submitted complete questionnaires were included in the analysis, and no completed questionnaire was excluded from the final dataset. The survey system did not retain sufficient information to determine how many individuals opened the survey but did not provide consent or discontinued it before completing the questionnaire. Therefore, the number and specific reasons for non-completion could not be reported.

The 320 complete questionnaires represented 6.8% of the 4675 TOTBİD members recorded in April 2026. This proportion represents a crude participation fraction and should not be interpreted as a conventional response rate because the investigators could not determine how many mailing-group subscribers successfully received, opened, or read the invitation. The number of physicians who actively declined participation was also unavailable.

The questionnaire did not collect names, national identification numbers, institutional identification numbers, email addresses, or other directly identifying information. The survey system was not technically cond to prevent the same individual from submitting more than one response. Because participation was fully anonymous, repeated participation could not be conclusively identified or excluded. Participants were instructed to complete the questionnaire only once. The inability to verify unique participation was considered a limitation of the recruitment procedure.

**Figure 1:**
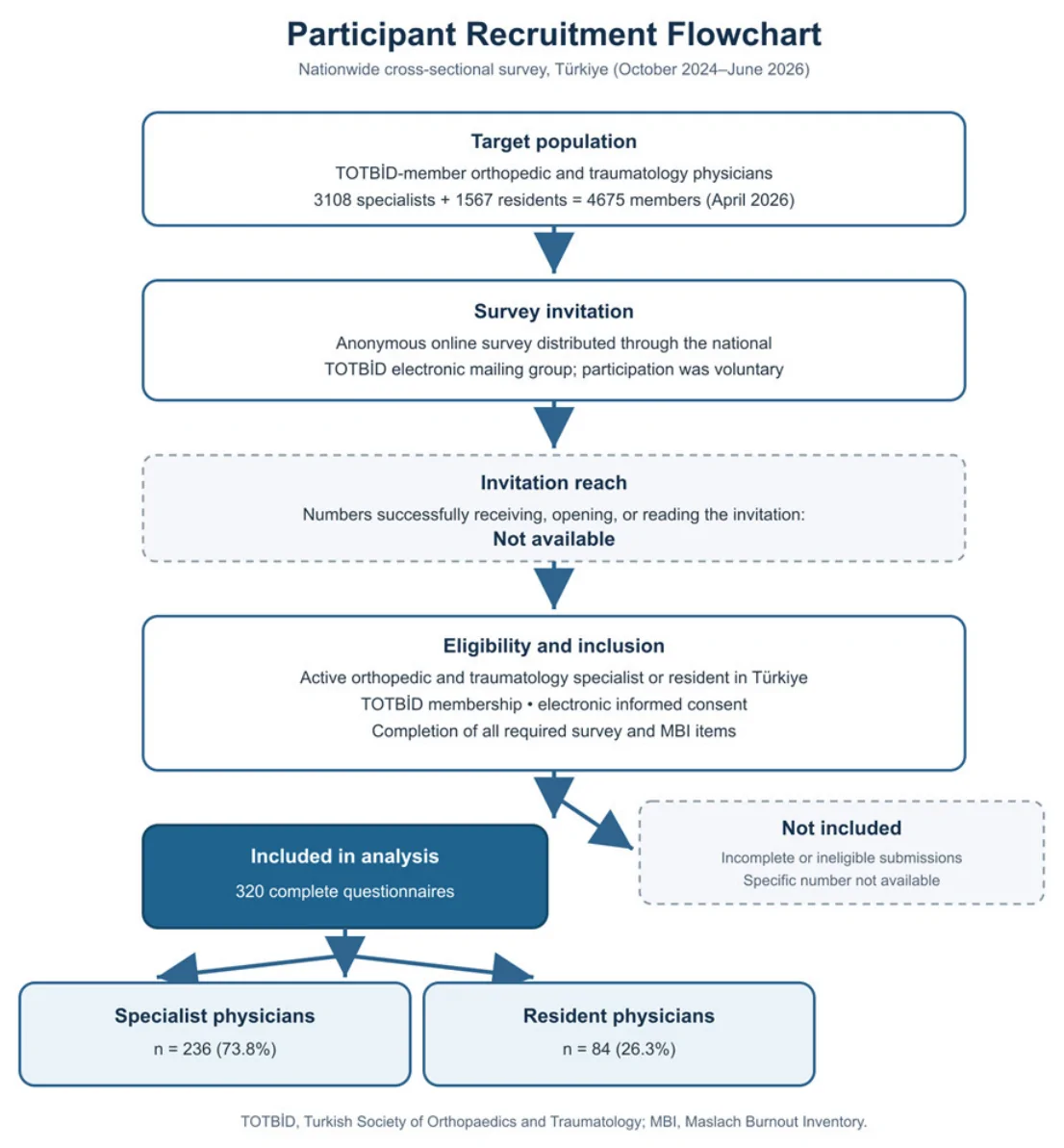
Participant recruitment and inclusion flowchart. TOTBİD, Turkish Society of Orthopedics and Traumatology; MBI, Maslach Burnout Inventory.

The participant recruitment and inclusion process is presented in Figure 1.

### 2.3. Sample-Size Adequacy

A formal a priori sample-size calculation was not performed before recruitment. The adequacy of the achieved sample was therefore evaluated in relation to established recommendations for multiple linear regression. The most extensively adjusted model included nine conceptual predictor groups represented by 22 regression parameters after categorical variables were dummy-coded: age group, marital status, professional status, institution type, practice location, daily patient volume, weekly working hours, monthly on-call duties, and monthly standby duties.

Green recommended a minimum sample size of (*n*\geq 50 + 8 m) for evaluating the overall multiple correlation, where (m) represents the number of predictors included in the model [21]. With 22 regression parameters, this criterion yielded a minimum recommended sample of 226 participants. The final sample of 320 exceeded this threshold. A sensitivity assessment conducted under a fixed-effects multiple-regression framework, with 22 predictors and a two-sided α level of 0.05, indicated that the achieved sample provided approximately 80% power to detect an overall effect of (f^2^\approx 0.07). The sample was therefore considered adequate for detecting small-to-moderate overall model effects. Nevertheless, statistical precision was expected to be lower for individual coefficients corresponding to categories with relatively few participants.

### 2.4. Demographic and Occupational Variables

The questionnaire consisted of a demographic and occupational information section followed by the burnout instrument. Demographic variables included age, sex, and marital status. Occupational variables included professional status, institution type, practice location, daily patient volume, weekly working hours, monthly on-call duties, and monthly standby duties.

Age was categorized as 24–30, 31–40, 41–50, 51–65, or ≥65 years. Marital status was categorized as married, single, or divorced. Professional status was classified as resident or specialist physician. Institution type was categorized as secondary-level public hospital, training and research hospital, university hospital, or other institution. Practice location was classified as district, provincial center, or metropolitan city.

Daily patient volume was categorized as 1–40, 41–80, 81–120, or >120 patients. Weekly working hours were categorized as 40–60, 61–80, 81–100, or >100 h. Monthly on-call duties were categorized as 1–3, 4–7, or 8–11, and monthly standby duties were categorized as 1–7, 8–14, or 15–21.

### 2.5. Burnout Assessment

Burnout was assessed using the Turkish adaptation of the 22-item Maslach Burnout Inventory–Human Services Survey for Medical Personnel. The original MBI conceptualizes burnout through three distinct dimensions: Emotional Exhaustion, Depersonalization, and Personal Accomplishment [1,2]. The Turkish version was adapted by Ergin for use among physicians and nurses [22]. Permission to use the Turkish adaptation was obtained from its adaptor before data collection. Further details regarding the administered Turkish MBI, including its response format, subscale composition, scoring procedure, internal consistency, and an English presentation of the items, are provided in the Supplementary Material.

Items were rated on a five-point Likert scale ranging from 0 (“Never”) to 4 (“Always”). EE scores ranged from 0 to 36, DP scores from 0 to 20, and PA scores from 0 to 32. Higher EE and DP scores indicate a less favorable burnout profile, whereas lower PA scores indicate reduced perceived professional accomplishment. The three dimensions were analyzed separately rather than combined into a single overall burnout score. Participants were therefore not dichotomously classified as “burned out” or “not burned out.”

Internal consistency was assessed separately for each MBI subscale using Cronbach’s alpha. Alpha coefficients of at least 0.70 were considered indicative of acceptable internal consistency.

### 2.6. Ethical Considerations

Ethical approval was obtained from the Harran University Clinical Research Ethics Committee before data collection (Approval No. HRÜ/24.14.30; 23 September 2024). The opening page of the survey provided information about the study objectives, voluntary participation, confidentiality, and the intended use of the data. Participants provided electronic informed consent before accessing the questionnaire.

No directly identifying information was collected. Survey data were stored and analyzed anonymously. The study was conducted in accordance with the principles of the Declaration of Helsinki.

### 2.7. Statistical Analysis

Continuous variables were summarized using means and standard deviations (SDs) and, where appropriate, medians and interquartile ranges (IQRs). Categorical variables were summarized using frequencies and percentages. The distributions of the MBI subscale scores were evaluated using histograms, Q–Q plots, skewness and kurtosis statistics, and the Shapiro–Wilk test. Because the subscale scores were bounded and derived from ordinal items, two of the three subscales showed statistically significant departures from normality, group sizes were unequal, and some categories included relatively few participants, nonparametric methods were used for unadjusted group comparisons and correlation analyses.

Burnout scores were compared between resident and specialist physicians using the Mann–Whitney U test, and rank-biserial correlations were calculated as effect-size estimates for these comparisons. Spearman’s rank correlation coefficients were used to examine monotonic associations between the ordered workload variables—daily patient volume, weekly working hours, monthly on-call duties, and monthly standby duties—and the EE, DP, and PA scores. Spearman correlations were also used to examine associations among the three MBI subscales. The internal consistency of each MBI subscale was evaluated using Cronbach’s alpha.

Separate multivariable linear regression models were fitted for EE, DP, and PA. The suitability of linear regression was evaluated on the basis of the distributions and diagnostic properties of the model residuals rather than the marginal distributions of the dependent variables alone. All prespecified demographic and occupational variables were entered simultaneously based on their theoretical relevance to workload, career stage, and working conditions. These variables included age group, marital status, professional status, institution type, practice location, daily patient volume, weekly working hours, monthly on-call duties, and monthly standby duties. Categorical variables were represented using indicator coding, with specialist physician as the reference category for professional status, the lowest category as the reference for each workload variable, and the most clinically appropriate category as the reference for the remaining variables. Sex was not included in the adjusted models because the very small number of female participants precluded stable coefficient estimation.

Regression assumptions and model adequacy were evaluated before the coefficients were interpreted. Linearity was assessed using residual-versus-fitted plots and the Rainbow test. Potential functional-form misspecification was examined using the Ramsey Regression Equation Specification Error Test (RESET). Homoscedasticity was evaluated using residual plots and the Breusch–Pagan test. Residual distributions were assessed using Q–Q plots, the Shapiro–Wilk test, and the Jarque–Bera test. Potentially influential observations were examined using Cook’s distance, leverage values, and studentized residuals. Multicollinearity among the independent variables was assessed using VIFs and tolerance statistics.

Because the Breusch–Pagan test indicated heteroscedasticity in the EE and DP models, HC3 heteroscedasticity-consistent standard errors were used for coefficient-level inference in all three regression models to ensure a consistent and conservative analytical approach. Regression findings were reported using unstandardized coefficients (B), HC3 robust 95% confidence intervals (CIs), standardized coefficients (β), and *p*-values. Partial (R^2^) values were calculated to describe the unique contribution of individual predictors. Overall model performance was summarized using (R^2^), adjusted (R^2^), the overall F statistic, and its corresponding *p*-value. All prespecified regression coefficients were retained and reported irrespective of statistical significance.

All analyses were performed using Python 3.12 with SciPy 1.17.0, statsmodels 0.14.6 statistical libraries. All statistical tests were two-sided, and *p* < 0.05 was considered statistically significant. No formal adjustment for multiple comparisons was applied because the predictors and outcomes were prespecified and the analyses were intended to estimate dimension-specific associations. Nevertheless, given the number of coefficients examined, secondary and exploratory findings were interpreted cautiously and in conjunction with confidence intervals, effect sizes, model diagnostics, category sizes, and the consistency of the observed associations. Statistical significance was not treated as the sole indicator of scientific or practical importance.

### 2.8. Assessment of Multicollinearity

Several demographic and occupational characteristics were expected to be interrelated. Multicollinearity among the independent variables was therefore evaluated before the multivariable regression coefficients were interpreted. Variance inflation factors (VIFs) and tolerance statistics were calculated for all predictors included in the adjusted models. VIF values below 5 and tolerance values above 0.20 were considered generally acceptable diagnostic indicators, although these thresholds were used as guidance rather than as absolute decision rules [23]. Values close to or exceeding these thresholds were examined in relation to category sizes, reference groups, and the stability of the corresponding coefficient estimates.

Correlations among EE, DP, and PA did not constitute multicollinearity because these subscales were entered as dependent variables in three separately estimated regression models. The multicollinearity assessment therefore focused exclusively on relationships among the demographic and occupational predictors. The range and maximum of the observed VIF values were reported with the regression diagnostics.

### 2.9. Assessment of Common Method Bias

Occupational characteristics and burnout outcomes were reported by the same participants through a single online questionnaire; therefore, the possibility of common method bias could not be excluded. Procedural measures used to reduce this risk included anonymous participation, the exclusion of directly identifying information, neutral question wording, the separation of demographic and occupational questions from the MBI items, and informing participants that there were no correct or incorrect responses.

As an exploratory statistical diagnostic, an unrotated factor analysis was conducted on the 22 MBI items, and the proportion of total variance explained by the first factor was examined using Harman’s single-factor approach [24]. A single factor accounting for the majority of the total variance would suggest the presence of a substantial common-method component within the MBI responses. However, because Harman’s test has limited sensitivity and does not directly evaluate shared-method variance between the categorical occupational variables and the burnout outcomes, it could not establish the absence of common method bias. Its findings were therefore interpreted cautiously and together with the procedural safeguards and acknowledged limitations of the single-source, self-report survey design [24].

## 3. Results

### 3.1. Participant Characteristics and Burnout Scores

A total of 320 orthopedic and traumatology physicians completed the survey. Of these, 236 (73.8%) were specialists and 84 (26.2%) were resident physicians. The demographic and occupational characteristics of the participants are presented in Table 1.

The mean Emotional Exhaustion (EE) score was 20.17 ± 6.83, with a median of 20.00 (interquartile range (IQR): 16.00–25.00). The mean Depersonalization (DP) score was 7.40 ± 3.58, with a median of 7.00 (IQR: 5.00–10.00), whereas the mean Personal Accomplishment (PA) score was 20.37 ± 4.19, with a median of 20.00 (IQR: 18.00–23.00). These values corresponded to 56.0%, 37.0%, and 63.6% of the theoretical maximum scores of the respective subscales.

Higher EE and DP scores and lower PA scores indicate a less favorable burnout profile. Because universally accepted diagnostic cutoffs have not been established for this occupational population and the Maslach Burnout Inventory is primarily interpreted dimensionally, the scores were not categorized as representing low, moderate, or high burnout. Instead, the means, medians, distributions, and theoretical score ranges are provided to contextualize the findings.

The internal consistency of the subscales was good for EE (Cronbach’s α = 0.89) and acceptable for DP (α = 0.74) and PA (α = 0.76). The EE scores did not significantly deviate from normality (Shapiro–Wilk *p* = 0.146), whereas the DP (*p* < 0.001) and PA scores (*p* = 0.016) showed statistically significant departures from normality. Given the bounded and ordinal-derived nature of the scores, the non-normality of two subscales, unequal group sizes, and several small category sizes, nonparametric tests were used for unadjusted group comparisons and correlation analyses.

#### Associations Between Workload and Burnout Dimensions

The correlations between workload indicators and burnout subscale scores are presented in Table 2. Daily patient volume was positively correlated with EE (Spearman’s ρ = 0.142, *p* = 0.011) and DP (ρ = 0.261, *p* < 0.001) and negatively correlated with PA (ρ = −0.112, *p* = 0.045). Weekly working hours were similarly associated with higher EE (ρ = 0.188, *p* < 0.001), higher DP (ρ = 0.254, *p* < 0.001), and lower PA (ρ = −0.157, *p* = 0.005).

The number of monthly on-call duties was positively associated with DP (ρ = 0.245, *p* < 0.001) and negatively associated with PA (ρ = −0.208, *p* < 0.001), but was not significantly associated with EE (ρ = 0.084, *p* = 0.132). Monthly standby duties showed a weak positive correlation with EE (ρ = 0.137, *p* = 0.015), whereas their correlations with DP and PA were not statistically significant. Overall, these associations were small, with the strongest workload-related correlation observed between daily patient volume and DP.

The burnout dimensions were moderately intercorrelated. EE was positively correlated with DP (ρ = 0.526, *p* < 0.001) and negatively correlated with PA (ρ = −0.445, *p* < 0.001). DP was also negatively correlated with PA (ρ = −0.546, *p* < 0.001).

### 3.2. Comparison of Resident and Specialist Physicians

Resident physicians had higher EE scores than specialists (21.56 ± 7.27 vs. 19.67 ± 6.62; Mann–Whitney U = 11,397.0, *p* = 0.041). However, the corresponding rank-biserial correlation indicated a small effect (r_rb = 0.150).

The difference in DP scores was more pronounced: residents had a mean DP score of 9.27 ± 3.83 compared with 6.74 ± 3.24 among specialists (U = 13,762.5, *p* < 0.001; r_rb = 0.388). Residents also had lower PA scores than specialists (18.81 ± 4.27 vs. 20.92 ± 4.02; U = 6881.0, *p* < 0.001; r_rb = −0.306). The effect sizes for the DP and PA comparisons were in the moderate range, suggesting that the differences were not limited to statistical significance alone.

Burnout subscale scores were compared between resident and specialist physicians using the Mann–Whitney U test, and the results are presented in Table 3.

### 3.3. Multivariable Regression Analyses

Separate multivariable linear regression models were fitted for EE, DP, and PA. All prespecified demographic and occupational variables were entered simultaneously based on their theoretical relevance to workload, career stage, and working conditions. The adjusted associations of professional status and the primary workload variables with each burnout dimension are presented in Table 4. Complete regression coefficients for all prespecified covariates, including statistically nonsignificant estimates, are provided in Supplementary Table S1, while model performance and diagnostic statistics are reported in Supplementary Table S2.

Because heteroscedasticity was detected in the EE model and was borderline in the DP model, heteroscedasticity-consistent HC3 standard errors, confidence intervals, and *p*-values were used for all three models. This approach also provided more conservative inference for the PA model, in which mild residual non-normality was observed.

The EE model was statistically significant, F(22, 297) = 2.37, *p* < 0.001, and explained 14.9% of the variance in EE scores (adjusted R^2^ = 0.086). After adjustment, seeing more than 120 patients per day was associated with a 4.22-point higher EE score compared with seeing 1–40 patients (B = 4.22, 95% CI: 0.59–7.85, standardized β = 0.172, *p* = 0.023). Working more than 100 h per week was associated with a 4.47-point higher EE score compared with working 40–60 h (B = 4.47, 95% CI: 1.12–7.83, standardized β = 0.215, *p* = 0.009). These differences corresponded to approximately 11.7% and 12.4% of the theoretical EE scale range, respectively, although their unique explained variance was small.

The DP model was statistically significant, F(22, 297) = 3.31, *p* < 0.001, with R^2^ = 0.197 and adjusted R^2^ = 0.138. Resident status was independently associated with a 2.14-point higher DP score (B = 2.14, 95% CI: 0.52–3.75, standardized β = 0.263, *p* = 0.010). Compared with participants who saw 1–40 patients per day, those seeing 41–80 patients (B = 1.57, 95% CI: 0.11–3.03, *p* = 0.035), 81–120 patients (B = 1.57, 95% CI: 0.00–3.13, *p* = 0.049), and more than 120 patients (B = 2.49, 95% CI: 0.42–4.55, *p* = 0.018) had higher DP scores. The largest patient-volume contrast represented approximately 12.4% of the theoretical DP scale range.

The PA model was also statistically significant, F(22, 297) = 2.04, *p* = 0.005, but had relatively limited explanatory power (R^2^ = 0.131; adjusted R^2^ = 0.067). Resident physicians had PA scores that were, on average, 1.92 points lower than those of specialists (B = −1.92, 95% CI: −3.52 to −0.33, standardized β = −0.203, *p* = 0.018). Participants reporting 15–21 monthly standby duties had a 2.38-point higher PA score than those reporting 1–7 duties (B = 2.38, 95% CI: 0.08–4.67, *p* = 0.042). Given the unexpected direction of this association, the small number of participants in this category, and the number of comparisons performed, this finding should be interpreted cautiously.

Some age-group coefficients reached statistical significance, particularly estimates involving participants aged ≥65 years. However, only three participants were included in this category; therefore, these estimates were considered unstable and were not interpreted substantively.

Taken together, the models explained a modest proportion of the variation in burnout scores. Individual predictors generally had small partial R^2^ values, indicating that burnout is unlikely to be attributable to a single demographic or workload factor.

**Figure 2:**
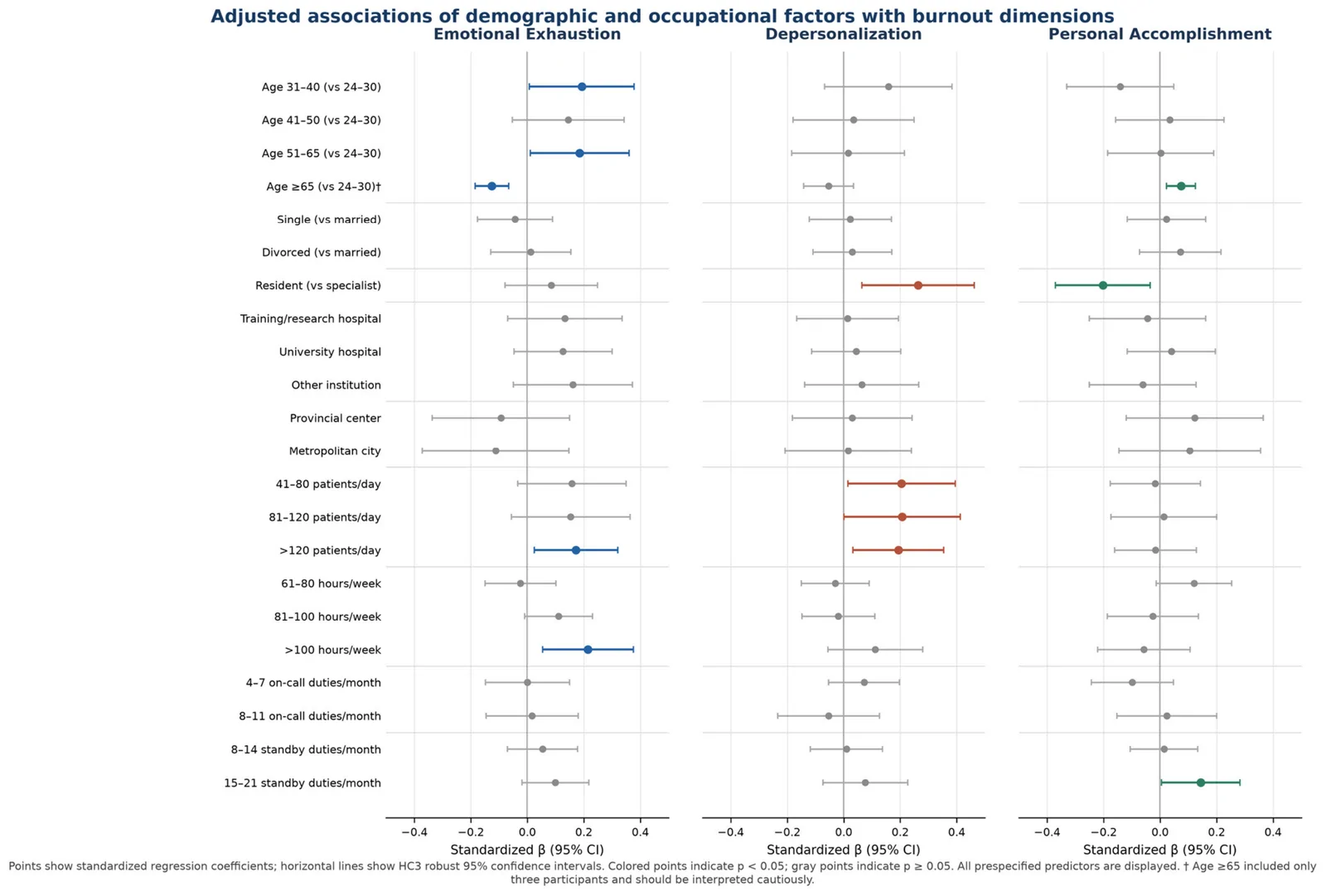
Adjusted associations of demographic and occupational factors with burnout dimensions. Points represent standardized regression coefficients, and horizontal lines represent 95% confidence intervals calculated using HC3 heteroscedasticity-consistent standard errors. Colored points indicate statistically significant associations (*p* < 0.05), whereas gray points indicate nonsignificant associations. Positive coefficients indicate higher subscale scores. Higher Emotional Exhaustion and Depersonalization scores and lower Personal Accomplishment scores represent a less favorable burnout profile. All prespecified predictors are displayed irrespective of statistical significance. The estimate for participants aged ≥65 years should be interpreted cautiously because this category included only three participants.

The adjusted associations of all prespecified demographic and occupational predictors with the three burnout dimensions are presented visually in Figure 2. Although several associations reached statistical significance, most standardized coefficients and their corresponding partial R^2^ values indicated small individual effects.

### 3.4. Regression Diagnostics

Detailed model-performance and regression-diagnostic results are presented in Supplementary Table S2. Rainbow tests provided no evidence of violation of the linearity assumption for the EE (*p* = 0.991), DP (*p* = 0.935), or PA models (*p* = 0.860). Ramsey RESET tests likewise showed no evidence of substantial functional-form misspecification.

The residuals of the EE and DP models were compatible with normality. Mild residual non-normality was observed in the PA model (Shapiro–Wilk *p* = 0.024; Jarque–Bera *p* = 0.002). The Breusch–Pagan test indicated heteroscedasticity in the EE model (*p* = 0.008) and evidence of heteroscedasticity in the DP model (*p* = 0.045), whereas no evidence of heteroscedasticity was found for PA (*p* = 0.838). Accordingly, HC3 robust inference was reported.

The maximum Cook’s distances were 0.040, 0.036, and 0.064 for the EE, DP, and PA models, respectively, and no observation had a Cook’s distance approaching 1. Three observations in the PA model had absolute studentized residuals greater than 3. These observations were retained because they represented plausible responses and no data-entry errors were identified. The overall conclusions were therefore interpreted in conjunction with robust standard errors and diagnostic findings.

Variance inflation factors were generally below 5. The highest VIF was 5.33 for metropolitan practice location, followed by 4.99 for provincial-center practice. These values were influenced by the small district reference category. Thus, there was no evidence of severe multicollinearity, although mild collinearity involving practice location should be acknowledged.

Because all variables were collected using the same self-report questionnaire, a Harman single-factor analysis was performed as an exploratory assessment of common method bias. The first unrotated factor accounted for 32.2% of the total variance, which was below the commonly used 50% criterion and did not indicate that a single factor dominated the covariance structure.

Nevertheless, the Harman test cannot exclude common method bias conclusively. The possibility of shared-method variance therefore remains a limitation of the self-report and cross-sectional data collection procedure.

Complete coefficient estimates for all prespecified predictors in the three multivariable regression models are presented in Supplementary Table S1.

### 3.5. Sensitivity Analysis Excluding Female Participants

A sensitivity analysis was conducted after excluding the six female participants (*n* = 314). The mean EE, DP, and PA scores were 20.25 ± 6.83, 7.40 ± 3.60, and 20.36 ± 4.21, respectively. The internal consistency coefficients remained essentially unchanged (EE: α = 0.888; DP: α = 0.741; PA: α = 0.759). The directions and statistical significance patterns of the workload correlations and resident–specialist comparisons were also preserved.

In the adjusted models, seeing more than 120 patients per day remained associated with higher EE (B = 3.89, 95% CI: 0.22–7.55, *p* = 0.038) and DP (B = 2.56, 95% CI: 0.46–4.66, *p* = 0.017), while working more than 100 h per week remained associated with higher EE (B = 4.36, 95% CI: 0.95–7.77, *p* = 0.012). Resident status remained associated with higher DP (B = 2.35, 95% CI: 0.72–3.97, *p* = 0.005) and lower PA (B = −2.05, 95% CI: −3.68 to −0.42, *p* = 0.014). Thus, excluding the female participants did not materially alter the principal findings. Complete sensitivity-analysis results are provided in Table 5.

## 4. Discussion

This study identified dimension-specific associations between occupational characteristics and burnout among orthopedic and traumatology physicians in Türkiye. Very high daily patient volume and prolonged weekly working hours were independently associated with higher Emotional Exhaustion (EE), increasing patient volume was associated with higher Depersonalization (DP), and resident status was associated with higher DP and lower Personal Accomplishment (PA). The contribution of these findings lies not simply in confirming that workload is related to burnout, but in demonstrating that workload and professional status have different adjusted relationships with the three burnout dimensions. These associations were observed after simultaneous adjustment for the measured demographic, institutional, and occupational characteristics. Nevertheless, the modest adjusted R^2^ and partial R^2^ values indicate that the measured variables explain only a limited proportion of individual differences in burnout.

The mean subscale scores should not be interpreted as evidence of a high prevalence of burnout. The Turkish adaptation of the MBI has demonstrated acceptable reliability [22], and historical normative data for Turkish healthcare personnel have been published [25]. However, these norms are approximately three decades old, were obtained from broader healthcare-worker populations, and are not specific to contemporary orthopedic and traumatology physicians or necessarily to the scoring configuration used in the present study. Furthermore, psychometric validity does not establish clinically meaningful diagnostic thresholds. Given the substantial heterogeneity in MBI versions, response scales, cutoff values, and definitions of burnout across the literature, the current scores are most appropriately interpreted as continuous dimensions rather than low-, moderate-, or high-burnout categories [2,5].

The associations between workload and EE were consistent with the expectation that sustained quantitative demands progressively consume emotional and physical resources. After adjustment, seeing more than 120 patients per day and working more than 100 h per week were each associated with an approximately 4-point increase in EE. Increasing patient volume was also associated with higher DP, suggesting that intensive clinical throughput may be related both to exhaustion and to greater psychological distancing from patients. This pattern is compatible with the Job Demands–Resources framework, although the present study did not directly measure job resources or test the mechanisms proposed by that model [26].

Previous findings concerning individual workload indicators have not been entirely consistent. Among Ecuadorian physicians, long working hours and work–family conflict were associated with higher EE, while shift work and work–family conflict were associated with higher DP [13]. Among anesthesia technologists, technicians, and trainees, occupational stress was associated with EE and DP, and night-shift work was associated with DP [27]. In contrast, Hean et al. found no significant differences in MBI scores between pharmacy residents with and without weekend staffing requirements, although EE increased over the residency year in the overall cohort [14]. Similarly, weekly working hours were not significantly associated with the MBI dimensions among Spanish implant-dentistry professionals [28]. These differences may reflect variation in professional responsibilities, workload intensity, autonomy, predictability, recovery opportunities, institutional support, and measurement methods. Therefore, hours worked or duties performed should not be assumed to represent the psychological burden of work equally across all professional settings.

The present findings refine previous evidence by showing that the associations also differed across burnout dimensions. Patient volume had a more consistent relationship with DP than weekly working hours or duty frequency, whereas very long working hours were most clearly associated with EE. Monthly on-call duties were associated with DP and PA in unadjusted analyses, but these relationships did not remain statistically significant after adjustment. This attenuation suggests that some unadjusted workload associations may reflect overlapping professional or institutional characteristics. It also emphasizes the importance of evaluating several workload indicators simultaneously rather than interpreting each indicator in isolation.

Residents had higher EE and DP and lower PA than specialists in the unadjusted comparisons. After adjustment, resident status remained associated with higher DP and lower PA, whereas the association with EE was no longer statistically significant. Higher burnout frequencies among orthopedic residents have been reported in Iran and Jordan [10,12], and Sargent et al. found burnout in 56% of residents compared with 28% of faculty members [17]. Wong et al. also concluded that orthopedic resident burnout is influenced by clinical, organizational, and work–home factors [15]. However, results have not been uniform. Simons et al. observed a higher burnout proportion among staff physicians in a small military orthopedic program, although a greater proportion of residents were classified as being at risk [16]. Differences in training programs, institutional environments, professional autonomy, sample sizes, and burnout definitions may account for this variability.

The adjusted results suggest that residency may be particularly relevant to the interpersonal and professional-efficacy dimensions of burnout. Higher DP could reflect psychological distancing in response to repeated clinical demands, limited control over scheduling, or hierarchical working conditions. Lower PA may be related to limited autonomy, frequent assessment, uncertainty about clinical competence, or reduced opportunity to observe long-term treatment outcomes. However, none of these mechanisms was directly measured. They should therefore be considered hypotheses for future research rather than established explanations of the observed associations.

The modest explanatory power of the regression models indicates that quantitative workload and demographic characteristics alone are insufficient to explain burnout. Institutional culture, leadership quality, staffing adequacy, professional autonomy, psychological safety, mentorship, team functioning, and participation in decision-making may influence how physicians experience comparable workloads. Shanafelt et al. reported that physicians’ evaluations of their immediate supervisors’ leadership qualities were independently associated with burnout and professional satisfaction [29]. This finding supports the interpretation that burnout is partly shaped by organizational conditions rather than representing solely an individual difficulty in coping with occupational demands.

Individual and interpersonal resources may also modify these relationships. Grit and resilience were inversely associated with burnout among Jordanian orthopedic surgeons [12]. Work–family conflict, psychological inflexibility, and loneliness were associated with unfavorable burnout dimensions among Ecuadorian physicians [13]. Social support, physical activity, mentorship, coping strategies, and work–life balance have also been identified as potentially relevant factors in orthopedic populations [10,15,17]. Because the present study did not assess these variables, it cannot determine whether they mediate or buffer the relationships between workload and burnout. Their omission may partly account for the limited proportion of variance explained by the models.

The demographic coefficients should be interpreted cautiously. Although some age coefficients reached statistical significance, the group aged ≥65 years included only three participants, making these estimates unstable. The positive association between 15–21 monthly standby duties and PA was also unexpected, involved a small subgroup, and explained little unique variance. It may reflect unmeasured seniority, differences in professional roles, self-selection, or chance arising from multiple comparisons. These exploratory findings should not form the basis of clinical or organizational recommendations. Other demographic characteristics are more appropriately presented as sample descriptors and adjustment variables than as principal findings.

### 4.1. Practical Implications

The practical implications should therefore extend beyond reducing working hours alone. Reviewing extreme patient volume, prolonged working weeks, staffing adequacy, scheduling practices, and opportunities for recovery represents a reasonable organizational starting point. For residents, structured mentorship, accessible supervision, protected educational time, participation in clinical decisions, and confidential psychological support may be particularly relevant. However, the cross-sectional findings do not establish that any specific intervention will reduce burnout.

Individual-focused strategies may complement, but should not replace, organizational changes. Khan et al. found that mindfulness, professional coaching, and structured peer-support programs were associated with statistically significant but generally modest improvements in EE, DP, and PA. The authors also noted limitations related to the small evidence base, heterogeneous intervention formats, and risk of bias [19]. An earlier systematic review and meta-analysis similarly found that both physician-directed and organization-directed interventions could reduce burnout [30]. These findings support multilevel approaches that address structural working conditions while also strengthening peer connection, coping resources, and access to psychological support.

### 4.2. Limitations

Several limitations should be considered. The cross-sectional design precludes establishing temporal direction or causality. Participation was voluntary, recruitment was conducted through the TOTBİD email network, and the number of physicians who received or opened the invitation could not be determined. Consequently, a conventional response rate could not be calculated and selection bias cannot be excluded. The survey platform did not technically prevent an individual from submitting more than one response.

All variables were self-reported, creating the possibility of recall, social-desirability, and common-method biases. Although Harman’s single-factor analysis did not indicate that one factor dominated the covariance structure, this procedure cannot conclusively exclude common-method bias [24]. The data-collection period extended from 2024 to 2026, and unmeasured temporal changes in individual workloads or institutional conditions may have introduced additional heterogeneity.

The predominance of male participants and the small numbers in several categories limit generalizability and statistical precision. Occupational exposures were recorded in broad categories rather than as continuous measurements. Finally, institutional culture, leadership, organizational support, autonomy, resilience, coping strategies, and work–life balance were not measured. The study therefore cannot determine the contribution of these potentially important organizational and personal resources.

Despite these limitations, the study provides dimension-specific evidence concerning burnout among orthopedic and traumatology physicians in Türkiye. Its principal contribution is the demonstration that patient volume, working hours, and professional status have different adjusted relationships with EE, DP, and PA. Future prospective and multicenter studies should combine objective workload indicators with measures of organizational culture, leadership, autonomy, work–life conflict, resilience, coping, and recovery. Such studies could clarify the mechanisms linking occupational demands to the individual dimensions of burnout and provide a stronger basis for evaluating targeted organizational and individual interventions.

## 5. Conclusions

The first hypothesis was partially supported. Very high daily patient volume and working more than 100 h per week were independently associated with higher Emotional Exhaustion. Higher daily patient volume was also associated with higher Depersonalization. However, weekly working hours were not independently associated with Depersonalization or Personal Accomplishment, and monthly on-call and standby duties did not show consistent adjusted associations with the three burnout dimensions. Thus, the findings support an association between selected indicators of high workload and specific burnout dimensions rather than a uniform relationship between all workload indicators and overall burnout.

The second hypothesis was also partially supported. After adjustment for demographic, institutional, and workload-related characteristics, resident physicians had higher Depersonalization and lower Personal Accomplishment scores than specialists. However, resident status was not independently associated with Emotional Exhaustion. These findings indicate that professional status is more strongly related to the interpersonal-detachment and professional-efficacy dimensions of burnout than to Emotional Exhaustion.

The sensitivity analysis excluding the six female participants produced a comparable pattern of findings, indicating that the principal results were not materially influenced by their inclusion. Nevertheless, the modest explanatory power of the regression models suggests that the measured demographic and workload characteristics account for only a limited proportion of the variation in burnout scores. Because of the cross-sectional design and voluntary non-probability sampling, the observed relationships should not be interpreted as causal or nationally representative. Prospective studies incorporating organizational culture, leadership, professional autonomy, institutional support, work–life balance, and objective workload measures are needed to confirm these dimension-specific associations.

## Figures and Tables

**Table 1:** Demographic and occupational characteristics of the participants (*n* = 320). Distribution and reliability of the burnout subscale scores.

**Characteristic**	**Category**								***n***	**%**
Sex	Male								313	97.8
	Female								6	1.9
	Prefer not to state								1	0.3
Age, years	24–30								68	21.2
	31–40								143	44.7
	41–50								60	18.8
	51–65								46	14.4
	≥65								3	0.9
Marital status	Married								256	80.0
	Single								52	16.2
	Divorced								12	3.8
Professional status	Specialist physician								236	73.8
	Resident physician								84	26.2
Institution type	Secondary-level state hospital								66	20.6
	Training and research hospital								151	47.2
	University hospital								42	13.1
	Other								61	19.1
Practice location	District								35	10.9
	Provincial center								144	45.0
	Metropolitan city								141	44.1
Daily patient volume	1–40								86	26.9
	41–80								101	31.6
	81–120								106	33.1
	>120								27	8.4
Weekly working hours	40–60								158	49.4
	61–80								85	26.6
	81–100								38	11.9
	>100								39	12.2
Monthly on-call duties	1–3								174	54.4
	4–7								114	35.6
	8–11								32	10.0
Monthly standby duties	1–7								247	77.2
	8–14								51	15.9
	15–21								22	6.9
**Subscale**	***n***	**Mean**	**SD**	**Median**	**IQR**	**Observed range**	**Scale maximum**	**Mean/maximum**	**Cronbach’s α**	**Shapiro–Wilk *p***
Emotional Exhaustion	320	20.17	6.83	20.00	16.00–25.00	2.00–36.00	36	56.0%	0.89	0.146
Depersonalization	320	7.40	3.58	7.00	5.00–10.00	0.00–18.00	20	37.0%	0.74	<0.001
Personal Accomplishment	320	20.37	4.19	20.00	18.00–23.00	5.00–31.00	32	63.6%	0.76	0.016

*Higher EE and DP scores and lower PA scores indicate a less favorable burnout profile. Scores were interpreted dimensionally; diagnostic low/moderate/high categories were not assigned.*

**Table 2:** Spearman correlations between workload indicators and burnout subscale scores.

Workload Indicator	Outcome	Spearman’s ρ	*p*
Daily patient volume	Emotional Exhaustion	0.142	0.011
	Depersonalization	0.261	<0.001
	Personal Accomplishment	−0.112	0.045
Weekly working hours	Emotional Exhaustion	0.188	<0.001
	Depersonalization	0.254	<0.001
	Personal Accomplishment	−0.157	0.005
Monthly on-call duties	Emotional Exhaustion	0.084	0.132
	Depersonalization	0.245	<0.001
	Personal Accomplishment	−0.208	<0.001
Monthly standby duties	Emotional Exhaustion	0.137	0.015
	Depersonalization	0.024	0.663
	Personal Accomplishment	0.101	0.072

**Table 3:** Comparison of burnout scores between resident and specialist physicians.

Outcome	Residents: Mean ± SD	Residents: Median (IQR)	Specialists: Mean ± SD	Specialists: Median (IQR)	Mann–Whitney U	*p*	Rank-Biserial r
Emotional Exhaustion	21.56 ± 7.27	22.00 (16.75–27.00)	19.67 ± 6.62	20.00 (15.00–24.00)	11,397.0	0.041	0.150
Depersonalization	9.27 ± 3.83	9.50 (7.00–12.00)	6.74 ± 3.24	6.00 (5.00–9.00)	13,762.5	<0.001	0.388
Personal Accomplishment	18.81 ± 4.27	19.00 (16.00–21.00)	20.92 ± 4.02	21.00 (18.75–24.00)	6881.0	<0.001	−0.306

*Residents: n = 84; specialists: n = 236. A positive rank-biserial correlation indicates higher scores among residents.*

**Table 4:** Adjusted associations of professional status and workload factors with burnout dimensions.

Outcome	Primary Predictor/Contrast	B	HC3 95% CI	Standardized β	*p*
Emotional Exhaustion	Resident vs. specialist	1.31	−1.23 to 3.84	0.084	0.311
	41–80 vs. 1–40 patients/day	2.31	−0.50 to 5.12	0.157	0.107
	81–120 vs. 1–40 patients/day	2.22	−0.83 to 5.27	0.153	0.153
	>120 vs. 1–40 patients/day	4.22	0.59 to 7.85	0.172	0.023
	61–80 vs. 40–60 h/week	−0.38	−2.32 to 1.56	−0.025	0.701
	81–100 vs. 40–60 h/week	2.33	−0.21 to 4.87	0.110	0.072
	>100 vs. 40–60 h/week	4.47	1.12 to 7.83	0.215	0.009
	4–7 vs. 1–3 on-call duties/month	0.00	−2.12 to 2.13	0.000	0.997
	8–11 vs. 1–3 on-call duties/month	0.38	−3.33 to 4.09	0.017	0.842
	8–14 vs. 1–7 standby duties/month	1.00	−1.32 to 3.31	0.053	0.398
	15–21 vs. 1–7 standby duties/month	2.68	−0.51 to 5.86	0.099	0.099
Depersonalization	Resident vs. specialist	2.14	0.52 to 3.75	0.263	0.010
	41–80 vs. 1–40 patients/day	1.57	0.11 to 3.03	0.204	0.035
	81–120 vs. 1–40 patients/day	1.57	0.00 to 3.13	0.207	0.049
	>120 vs. 1–40 patients/day	2.49	0.42 to 4.55	0.193	0.018
	61–80 vs. 40–60 h/week	−0.25	−1.22 to 0.73	−0.030	0.619
	81–100 vs. 40–60 h/week	−0.21	−1.63 to 1.21	−0.019	0.770
	>100 vs. 40–60 h/week	1.22	−0.61 to 3.05	0.112	0.191
	4–7 vs. 1–3 on-call duties/month	0.54	−0.40 to 1.47	0.072	0.259
	8–11 vs. 1–3 on-call duties/month	−0.64	−2.78 to 1.51	−0.053	0.560
	8–14 vs. 1–7 standby duties/month	0.09	−1.15 to 1.33	0.009	0.885
	15–21 vs. 1–7 standby duties/month	1.07	−1.05 to 3.20	0.076	0.320
Personal Accomplishment	Resident vs. specialist	−1.92	−3.52 to −0.33	−0.203	0.018
	41–80 vs. 1–40 patients/day	−0.15	−1.59 to 1.29	−0.017	0.834
	81–120 vs. 1–40 patients/day	0.11	−1.55 to 1.77	0.013	0.892
	>120 vs. 1–40 patients/day	−0.24	−2.42 to 1.94	−0.016	0.827
	61–80 vs. 40–60 h/week	1.13	−0.13 to 2.39	0.120	0.078
	81–100 vs. 40–60 h/week	−0.34	−2.42 to 1.74	−0.026	0.750
	>100 vs. 40–60 h/week	−0.74	−2.83 to 1.35	−0.058	0.485
	4–7 vs. 1–3 on-call duties/month	−0.86	−2.12 to 0.41	−0.098	0.184
	8–11 vs. 1–3 on-call duties/month	0.33	−2.13 to 2.78	0.024	0.792
	8–14 vs. 1–7 standby duties/month	0.16	−1.21 to 1.52	0.014	0.823
	15–21 vs. 1–7 standby duties/month	2.38	0.08 to 4.67	0.144	0.042

*All models were additionally adjusted for age, marital status, institution type, and practice location. HC3 heteroscedasticity-consistent confidence intervals and p-values are reported. Complete coefficients for all prespecified variables are provided in Supplementary Table S1.*

**Table 5:** Sensitivity Analyses After Excluding the Six Female Participants. Comparison of the full sample (*n* = 320) with the sensitivity sample excluding female participants (*n* = 314).

**Panel (A). Burnout subscale distributions and internal consistency**										
**Subscale**	**Full mean ± SD**		**Full median (IQR)**		**Full α**	**Sensitivity mean ± SD**		**Sensitivity median (IQR)**		**Sensitivity α**
Emotional Exhaustion	20.17 ± 6.83		20.00 (16.00–25.00)		0.888	20.25 ± 6.83		20.00 (16.00–25.00)		0.888
Depersonalization	7.40 ± 3.58		7.00 (5.00–10.00)		0.736	7.40 ± 3.60		7.00 (5.00–10.00)		0.741
Personal Accomplishment	20.37 ± 4.19		20.00 (18.00–23.00)		0.757	20.36 ± 4.21		20.50 (18.00–23.00)		0.759
**Panel (B). Resident–specialist comparisons**										
**Outcome**	**Full residents**	**Full specialists**	**Full U**	**Full p**	**Full r_rb**	**Sensitivity residents**	**Sensitivity specialists**	**Sensitivity U**	**Sensitivity p**	**Sensitivity r_rb**
Emotional Exhaustion	21.56 ± 7.27	19.67 ± 6.62	11,397.0	0.041	0.150	21.62 ± 7.33	19.77 ± 6.59	10,920.5	0.046	0.148
Depersonalization	9.27 ± 3.83	6.74 ± 3.24	13,762.5	<0.001	0.388	9.35 ± 3.84	6.71 ± 3.24	13,362.0	<0.001	0.405
Personal Accomplishment	18.81 ± 4.27	20.92 ± 4.02	6881.0	<0.001	−0.306	18.76 ± 4.30	20.93 ± 4.04	6519.0	<0.001	−0.315

## Data Availability

The anonymized datasets generated and analyzed during the current study are available from the corresponding author upon reasonable request, subject to ethical and confidentiality considerations.

## Supplementary Materials

The following supporting information can be downloaded at: https://www.mdpi.com/article/10.3390/healthcare14172696/s1, Supplementary Table S1. Complete multivariable linear regression models for burnout subscales; Supplementary Table S2. Model performance and regression diagnostics; Supplementary Material: Turkish Maslach Burnout Inventory: Items, Scoring, and Internal Consistency.

## Abbreviations

CI	Confidence Interval
DP	Depersonalization
EE	Emotional Exhaustion
HC3	Heteroscedasticity-Consistent Covariance Matrix Estimator, Type 3
IQR	Interquartile Range
MBI	Maslach Burnout Inventory
MBI-HSS	Maslach Burnout Inventory–Human Services Survey
PA	Personal Accomplishment
SD	Standard Deviation
TOTBİD	Turkish Society of Orthopaedics and Traumatology
VIF	Variance Inflation Factor
