# Supplementary material for: Burnout Dimensions and Associated Factors Among Orthopedic and Traumatology Physicians: A Cross-Sectional Study from Türkiye
Source: Healthcare (Basel). 2026 Aug 24;14(17):2696. doi: 10.3390/healthcare14172696 (PMC13564569; doi:10.3390/healthcare14172696)
Supplement: Supplementary file 1 [file healthcare-14-02696-s001.zip › healthcare-4502972-supplementary material.pdf]

SUPPLEMENTARY MATERIAL

Turkish Maslach Burnout Inventory: Items, Scoring, and Internal Consistency

English presentation of the Turkish version administered in the study

Instrument and Response Format

Burnout was assessed using the Turkish adaptation of the 22-item Maslach Burnout Inventory–Human Services Survey for Medical Personnel. Participants indicated how frequently they experienced each statement using a five-point response scale: Never (0), Rarely (1), Sometimes (2), Often (3), and Always (4). The English wording below is a direct rendering of the Turkish items administered in this study for supplementary reporting.

Subscales and Internal Consistency

Cronbach's alpha was calculated separately for each theoretically defined subscale. Higher EE and DP scores indicate a less favorable burnout profile, whereas lower PA scores indicate lower perceived professional accomplishment. A single alpha coefficient was not calculated across all 22 items because the instrument comprises three distinct dimensions.

Supplementary Table. Internal consistency of the MBI subscales (N = 320)

Subscale	Abbreviation	Item numbers	No. of items	Cronbach's α
Emotional Exhaustion	EE	1, 2, 3, 6, 8, 13, 14, 16, 20	9	0.89
Depersonalization	DP	5, 10, 11, 15, 22	5	0.74
Personal Accomplishment	PA	4, 7, 9, 12, 17, 18, 19, 21	8	0.76

Note: Cronbach's alpha is a scale-level reliability coefficient, not an item-level statistic. Item-level diagnostics such as corrected item–total correlations and Cronbach's alpha if item deleted require the participant-level responses to all 22 items and are therefore not reported here.

Scoring

Subscale scores were obtained by summing the relevant item responses. The theoretical score ranges were 0–36 for EE, 0–20 for DP, and 0–32 for PA. The three subscales were analyzed separately and were not combined into a single overall burnout score. Participants were not classified using low-, moderate-, or high-burnout thresholds.

Permission and use note. The Turkish adaptation was used with permission obtained from its adaptor before data collection. This supplementary presentation documents the items administered in the study and does not replace the licensed instrument or its administration manual.

Inventory Items

Supplementary Table. English presentation and subscale allocation of the 22 Turkish MBI items

Item	Subscale	Statement
1	EE	I feel emotionally drained from my work.
2	EE	I feel emotionally exhausted when I return home from work.
3	EE	When I get up in the morning, I feel that I cannot face another day at this job.
4	PA	I can readily understand how my patients feel.
5	DP	I feel that I treat some patients as if they were not human beings.
6	EE	Working with people all day is very demanding for me.
7	PA	I find the most appropriate solutions to my patients’ problems.
8	EE	I feel burned out by my work.
9	PA	I feel that my work makes a positive contribution to other people’s lives.
10	DP	I have become more insensitive toward people since I started this job.
11	DP	I am concerned that this job is making me emotionally harder.
12	PA	I feel capable of accomplishing many things.
13	EE	I feel frustrated by my job.
14	EE	I feel that I work too hard in my job.
15	DP	I do not really care what happens to some of my patients.
16	EE	Working directly with people places a great strain on me.
17	PA	I am able to create a relaxed atmosphere with my patients.
18	PA	I feel refreshed after working closely with people.
19	PA	I have accomplished many worthwhile things in this job.
20	EE	I feel that I have reached the end of my capacity.
21	PA	I deal calmly with emotional problems in my work.
22	DP	I feel that some patients blame me for some of their problems.
