# Supplementary material for: Burnout Dimensions and Associated Factors Among Orthopedic and Traumatology Physicians: A Cross-Sectional Study from Türkiye
Source: Healthcare (Basel). 2026 Aug 24;14(17):2696. doi: 10.3390/healthcare14172696 (PMC13564569; doi:10.3390/healthcare14172696)
Supplement: Supplementary file 1 [file healthcare-14-02696-s001.zip › healthcare-4502972-supplementary-table s1 & s2.pdf]

Supplementary Table S1. Complete multivariable linear regression models for burnout subscales

Outcome	Predictor/contrast	B	Robust SE	95% CI	Standardized β	Robust t	p	Partial R²	VIF
Emotional Exhaustion	Intercept	14.46	2.21	10.12 to 18.81		6.548	<0.001	0.109	
	Age 31–40 vs. 24–30	2.64	1.29	0.09 to 5.19	0.192	2.041	0.042	0.012	3.70
	Age 41–50 vs. 24–30	2.53	1.76	-0.93 to 5.98	0.145	1.439	0.151	0.007	3.60
	Age 51–65 vs. 24–30	3.60	1.72	0.20 to 6.99	0.185	2.086	0.038	0.012	3.22
	Age ≥65 vs. 24–30	-8.89	2.13	-13.09 to -4.70	-0.126	-4.174	<0.001	0.015	1.25
	Single vs. married	-0.81	1.25	-3.27 to 1.65	-0.044	-0.645	0.520	0.002	1.43
	Divorced vs. married	0.43	2.59	-4.66 to 5.52	0.012	0.166	0.869	0.000	1.09
	Resident vs. specialist	1.31	1.29	-1.23 to 3.84	0.084	1.015	0.311	0.003	3.07
	Training/research vs. state hospital	1.82	1.41	-0.95 to 4.59	0.133	1.290	0.198	0.006	3.35
	University vs. state hospital	2.55	1.79	-0.96 to 6.06	0.126	1.428	0.154	0.007	2.57
	Other institution vs. state hospital	2.79	1.86	-0.87 to 6.46	0.161	1.502	0.134	0.008	4.01
	Provincial center vs. district	-1.29	1.70	-4.63 to 2.06	-0.094	-0.757	0.449	0.002	4.99
	Metropolitan city vs. district	-1.54	1.81	-5.11 to 2.02	-0.112	-0.851	0.395	0.003	5.33
	41–80 vs. 1–40 patients/day	2.31	1.43	-0.50 to 5.12	0.157	1.618	0.107	0.009	3.07
	81–120 vs. 1–40 patients/day	2.22	1.55	-0.83 to 5.27	0.153	1.434	0.153	0.008	3.64
	>120 vs. 1–40 patients/day	4.22	1.84	0.59 to 7.85	0.172	2.289	0.023	0.016	2.18
	61–80 vs. 40–60 hours/week	-0.38	0.99	-2.32 to 1.56	-0.025	-0.384	0.701	0.000	1.47
	81–100 vs. 40–60 hours/week	2.33	1.29	-0.21 to 4.87	0.110	1.804	0.072	0.009	1.58
	>100 vs. 40–60 hours/week	4.47	1.71	1.12 to 7.83	0.215	2.623	0.009	0.029	1.84

	4–7 vs. 1–3 on-call duties/month	0.00	1.08	-2.12 to 2.13	0.000	0.003	0.997	0.000	1.66
	8–11 vs. 1–3 on-call duties/month	0.38	1.88	-3.33 to 4.09	0.017	0.200	0.842	0.000	1.99
	8–14 vs. 1–7 standby duties/month	1.00	1.18	-1.32 to 3.31	0.053	0.847	0.398	0.003	1.20
	15–21 vs. 1–7 standby duties/month	2.68	1.62	-0.51 to 5.86	0.099	1.655	0.099	0.009	1.24

Supplementary Table S1 continues on the following page.

Supplementary Table S1 (continued). Complete multivariable linear regression models for burnout subscales

Outcome	Predictor/contrast	B	Robust SE	95% CI	Standardized β	Robust t	p	Partial R^2	VIF
Depersonalization	Intercept	4.35	1.36	1.66 to 7.03		3.188	0.002	0.041	
	Age 31–40 vs. 24–30	1.13	0.82	-0.48 to 2.75	0.158	1.380	0.169	0.008	3.70
	Age 41–50 vs. 24–30	0.32	1.00	-1.64 to 2.28	0.035	0.320	0.749	0.000	3.60
	Age 51–65 vs. 24–30	0.16	1.03	-1.88 to 2.19	0.015	0.151	0.880	0.000	3.22
	Age ≥65 vs. 24–30	-2.00	1.66	-5.28 to 1.27	-0.054	-1.204	0.230	0.003	1.25
	Single vs. married	0.23	0.71	-1.18 to 1.63	0.023	0.316	0.752	0.000	1.43
	Divorced vs. married	0.57	1.33	-2.05 to 3.19	0.030	0.428	0.669	0.001	1.09
	Resident vs. specialist	2.14	0.82	0.52 to 3.75	0.263	2.605	0.010	0.027	3.07
	Training/research vs. state hospital	0.10	0.65	-1.19 to 1.39	0.014	0.150	0.881	0.000	3.35
	University vs. state hospital	0.47	0.85	-1.20 to 2.13	0.044	0.551	0.582	0.001	2.57
	Other institution vs. state hospital	0.58	0.93	-1.25 to 2.41	0.064	0.620	0.535	0.001	4.01
	Provincial center vs. district	0.22	0.77	-1.31 to 1.74	0.030	0.278	0.781	0.000	4.99
	Metropolitan city vs. district	0.11	0.82	-1.50 to 1.73	0.016	0.140	0.889	0.000	5.33
	41–80 vs. 1–40 patients/day	1.57	0.74	0.11 to 3.03	0.204	2.117	0.035	0.017	3.07
	81–120 vs. 1–40 patients/day	1.57	0.79	0.00 to 3.13	0.207	1.973	0.049	0.014	3.64
	>120 vs. 1–40 patients/day	2.49	1.05	0.42 to 4.55	0.193	2.371	0.018	0.021	2.18
	61–80 vs. 40–60 hours/week	-0.25	0.49	-1.22 to 0.73	-0.030	-0.498	0.619	0.001	1.47
	81–100 vs. 40–60 hours/week	-0.21	0.72	-1.63 to 1.21	-0.019	-0.292	0.770	0.000	1.58
	>100 vs. 40–60 hours/week	1.22	0.93	-0.61 to 3.05	0.112	1.310	0.191	0.008	1.84
	4–7 vs. 1–3 on-call duties/month	0.54	0.47	-0.40 to 1.47	0.072	1.132	0.259	0.004	1.66

	8–11 vs. 1–3 on-call duties/month	-0.64	1.09	-2.78 to 1.51	-0.053	-0.584	0.560	0.002	1.99
	8–14 vs. 1–7 standby duties/month	0.09	0.63	-1.15 to 1.33	0.009	0.145	0.885	0.000	1.20
	15–21 vs. 1–7 standby duties/month	1.07	1.08	-1.05 to 3.20	0.076	0.997	0.320	0.006	1.24

Supplementary Table S1 continues on the following page.

Supplementary Table S1 (continued). Complete multivariable linear regression models for burnout subscales

Outcome	Predictor/contrast	B	Robust SE	95% CI	Standardized β	Robust t	p	Partial R²	VIF
Personal Accomplishment	Intercept	20.53	1.35	17.86 to 23.19		15.156	<0.001	0.392	
	Age 31–40 vs. 24–30	-1.18	0.81	-2.78 to 0.41	-0.141	-1.466	0.144	0.006	3.70
	Age 41–50 vs. 24–30	0.37	1.04	-1.68 to 2.42	0.034	0.352	0.725	0.000	3.60
	Age 51–65 vs. 24–30	0.02	1.14	-2.21 to 2.26	0.002	0.020	0.984	0.000	3.22
	Age ≥65 vs. 24–30	3.20	1.13	0.98 to 5.43	0.074	2.832	0.005	0.005	1.25
	Single vs. married	0.25	0.80	-1.33 to 1.82	0.022	0.311	0.756	0.000	1.43
	Divorced vs. married	1.57	1.61	-1.60 to 4.74	0.071	0.977	0.329	0.005	1.09
	Resident vs. specialist	-1.92	0.81	-3.52 to -0.33	-0.203	-2.379	0.018	0.015	3.07
	Training/research vs. state hospital	-0.37	0.88	-2.10 to 1.36	-0.044	-0.423	0.673	0.001	3.35
	University vs. state hospital	0.49	0.98	-1.43 to 2.42	0.040	0.503	0.615	0.001	2.57
	Other institution vs. state hospital	-0.66	1.02	-2.66 to 1.35	-0.062	-0.643	0.520	0.001	4.01
	Provincial center vs. district	1.03	1.04	-1.01 to 3.06	0.122	0.990	0.323	0.003	4.99
	Metropolitan city vs. district	0.89	1.07	-1.22 to 3.00	0.105	0.827	0.409	0.002	5.33
	41–80 vs. 1–40 patients/day	-0.15	0.73	-1.59 to 1.29	-0.017	-0.210	0.834	0.000	3.07
	81–120 vs. 1–40 patients/day	0.11	0.84	-1.55 to 1.77	0.013	0.136	0.892	0.000	3.64
	>120 vs. 1–40 patients/day	-0.24	1.11	-2.42 to 1.94	-0.016	-0.218	0.827	0.000	2.18
	61–80 vs. 40–60 hours/week	1.13	0.64	-0.13 to 2.39	0.120	1.768	0.078	0.011	1.47
	81–100 vs. 40–60 hours/week	-0.34	1.06	-2.42 to 1.74	-0.026	-0.319	0.750	0.000	1.58
	>100 vs. 40–60 hours/week	-0.74	1.06	-2.83 to 1.35	-0.058	-0.699	0.485	0.002	1.84

	4–7 vs. 1–3 on-call duties/month	-0.86	0.64	-2.12 to 0.41	-0.098	-1.332	0.184	0.007	1.66
	8–11 vs. 1–3 on-call duties/month	0.33	1.25	-2.13 to 2.78	0.024	0.264	0.792	0.000	1.99
	8–14 vs. 1–7 standby duties/month	0.16	0.69	-1.21 to 1.52	0.014	0.224	0.823	0.000	1.20
	15–21 vs. 1–7 standby duties/month	2.38	1.17	0.08 to 4.67	0.144	2.039	0.042	0.019	1.24

All prespecified predictors are shown irrespective of statistical significance. B, SE, confidence intervals, t statistics, and p values use HC3 heteroscedasticity-consistent inference. Reference categories are stated in the contrasts.

Supplementary Table S2. Model performance and regression diagnostics

Outcome	R^2	Adjusted R^2	F (df)	Model p	RMSE	Rainbow p	RESET p	Breusch–Pagan p	Residual Shapiro p	Jarque–Bera p	Maximum Cook's D	Maximum leverage	Maximum |studentized residual|
Emotional Exhaustion	0.149	0.086	2.370 (22, 297)	<0.001	6.531	0.991	0.136	0.008	0.702	0.556	0.040	0.363	2.895
Depersonalization	0.197	0.138	3.312 (22, 297)	<0.001	3.323	0.935	0.798	0.045	0.236	0.419	0.036	0.363	2.778
Personal Accomplishment	0.131	0.067	2.035 (22, 297)	0.005	4.044	0.860	0.677	0.838	0.024	0.002	0.064	0.363	3.871

HC3 robust inference was used because the Breusch–Pagan test indicated heteroscedasticity in the EE and DP models. The PA model showed mild residual non-normality.
